# Investigating gunshot wounds in charred bone with XRF spectroscopy: a technical note

**DOI:** 10.1007/s00414-024-03274-4

**Published:** 2024-06-20

**Authors:** Letizia Bonizzoni, Debora Mazzarelli, Lorenzo Franceschetti, Chiara Vitali, Alberto Amadasi, Cristina Cattaneo

**Affiliations:** 1 Department of Physics Aldo Pontremoli, University of Milan, Milan, Italy; 2 LABANOF, Laboratory of Forensic Anthropology and Odontology, Institute of Legal Medicine, Department of Biomedical Sciences for Health, University of Milan, Milan, Italy; 3 Institute of Legal Medicine and Forensic Sciences, Charité-Universitätsmedizin Berlin, Turmstr21 (Haus M) 10559 Berlin, Germany; 4 Institute of Legal Medicine, Department of Biomedical Sciences for Health, University of Milan, Milan, Italy

**Keywords:** Gunshot residues, X-Ray fluorescence, Burnt remains, Charred bones, Cremation

## Abstract

The analysis of traces of injuries can be difficult in cases of charred human remains since the alteration and fragmentation are high. The aim of this study is to explore the use of X-Ray Fluorescence (XRF) technique as a screening tool for detecting and analyzing gunshot residues (GSR) on cremated and highly fragmented materials, as it is a technique that allows for fast qualitative investigations without altering the sample or requiring sample preparation. The study was carried out on two steps: firstly, on completed skeletonized bones to verify if GSR survive to burning; secondly, we considered a more realistic situation, in which soft tissues were present before the shooting. To this aim, nine adult bovine ribs, four retaining soft tissue, five completely skeletonized, were subjected to a shooting test using two types of 9 mm projectiles (jacketed and unjacketed bullets). The ribs were then burnt until complete calcination in an electric furnace. The entry wound of each rib was analyzed using XRF, revealing traces of GSR. The XRF analysis showed that all samples, except for one, contain Pb and/or Sb near the lesion. Furthermore, the samples hit by unjacketed bullets had a more significant presence of Pb in macroscopic yellow areas, which persisted when moving away from the gunshot. These findings could pave the way for the use of XRF technology, mostly when a fast and immediate scan must be done on osteologic materials by a conservative method.

## Introduction

In forensic pathology, fundamental is the research of gunshot residues in order to identify the type of weapon, the manner of death, the shooting distance and the crime’s dynamics. Gunshot residues (GSR) are usually detected in entrance wounds in gunshot lesions and derive from burnt and unburnt components that are involved in the shot such as the primer, the propulsive charge, the bullet, the cartridge case and the firearm [1–4].

When a firearm is used, these residues can be detected close to the firearm (such as the shooter’s hand or the gunshot entrance wound when the shot is fired at short distance) or within the tissues if the projectile has perforated them [5–8]. Over the years, several studies have been performed on the detection of GSR on many types of materials and by various methods, in order to give an unambiguous definition of “gunshot residues” and to evaluate which procedures are the most reliable for their recognition and analysis [9, 10]. At the state of the art, the association among lead (Pb), barium (Ba), and antimony (Sb) is considered as unique for GSR particles, while associations of two of them (like Pb–Ba, Pb–Sb, and Sb–Ba) are considered only as characteristic GSR particles. In fact, these three elements, in the form of lead styphnate, barium nitrate, and antimony sulfide, are combined in a single application for one product: the mix in the primer cap of a cartridge casing. Moreover, GSR primer particles typically show a characteristic spherical shape: this particle morphology, when combined with the elemental composition, makes GSR quite distinct from many environmental particulates [11–13].

In this paper we focus on the survival of GSR in cremated bones, to face the scenario in which fire is hypothetically used to try deleting the evidence on the corpse. Previous studies successfully used scanning electron microscopy coupled with energy dispersive X-ray analysis (SEM-EDX) [6] and radiological experiments [7] to investigate cremated bone. In these challenging cases, in fact, bodies subjected to the destructive action of heat can reach a high level of fragmentation (in the order of thousands of bone fragments) that would be impossible to analyse through the SEM due to the reduced flexibility of the instrument. An attempt to use a non-destructive, less expensive, and quicker method was made by means of Inductively Plasma Optical Emission Spectroscopy (ICP-OES) [14]: in that case, the specimens were taken from a 3 cm concentric area of the entrance wound with a cotton swab soaked on ultrapure nitric acid. In the present research, we used an X-ray fluorescence (XRF) device directly on the bones without any further treatment of the samples. XRF method is based on the interaction of X-ray radiation with atoms. The result is an emission of characteristic fluorescence X-ray radiation with unique energy for a particular element: this emission is the analytical signal registered in XRF [13]. XRF analysis is one of the popular methods of chemical analysis as it allows for non-destructive and express quantification of several elements simultaneously without complex sample pretreatment. XRF has found wide application in geochemistry, material science, archeology, etc. [7–12], but is not widely used in the forensic field, even though some recent applications have been published [15–17], also regarding GSR [18, 19].

The aim of this study is to verify the potential of the XRF device in detecting GSR in charred remains, firstly, on completed skeletonized bones to verify if GSR survive to burning; secondly, we considered a more realistic situation, in which soft tissues were present before the shooting. The ultimate purpose is to suggest the use of this instrument for screening in cases of destruction of a corpse by fire, even in cases where no morphological signs of the passage of a bullet are clearly visible.

## Materials and methods

In this study, nine adult bovine ribs, 20 to 27 cm in length and 2–5 cm thick, were used: four were still covered by soft tissue (mainly muscle) and were named “dressed” (“D”); the other five ribs underwent maceration in boiling water and were completely skeletonized and were named “naked” (“N”). Materials are similar to those used in previous study for radiology, SEM-EDX and ICP-OES [5, 6, 14].

**Fig. 1:**
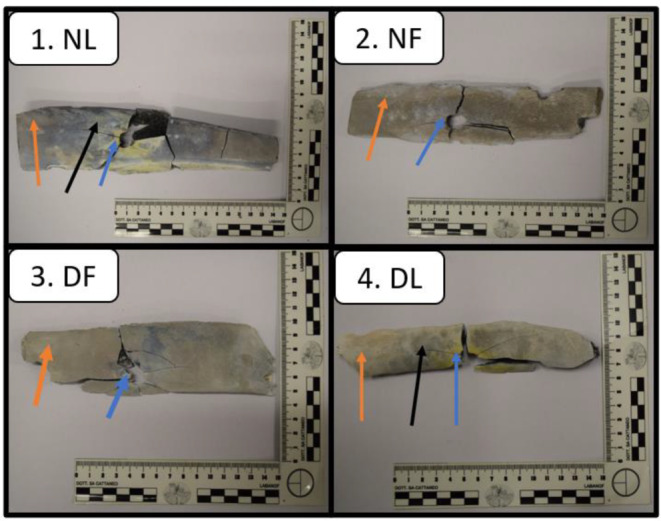
Examples for each charred rib group are reported. The arrows indicate the distance of the XFR analysis points from the gunshot hole: the blue ones indicate analysis close to the hole, the orange ones away from the hole, the black ones at an intermediate distance from the hole

All samples were shot at a firing ground. A “Beretta type 98 FS” (series 92) caliber 9 mm was the weapon used for our purpose. Two different kinds of projectile were used: “Magtech-cbc” LRN projectiles (with unjacketed lead bullets) and “Fiocchi” 123 projectiles (with full metal-jacketed bullets). All nine ribs were shot with a single bullet, at near-contact range. In consideration of the two types of ribs and the two type of projectiles, our samples were divided into four groups so named: (1) NF (“naked ribs, full metal-jacketed bullet), (2) NL (“naked” ribs, lead unjacketed bullet, (3) DF (“dressed” ribs, full-metal jacketed bullet), (4) DL (“dressed”, lead unjacketed bullet). Figure 1 shows the four-group sample. Each specimen was placed in a plastic bag and stored in a freezer. Then, the ribs underwent a “charring cycle” in an electric oven trying to simulate the carbonization process in human bodies. The complete cycle lasted 24 h; during the first 12 h, the samples were heated at 800°C, whereas in the latter 12 h, the samples were left to cool. Every sample reached the morphological features of “calcined bone” without any residual fragments of soft tissue as a result of the high temperatures used. The oven used to heat samples was an electric industrial machine “Vega” s.r.l.”, model “Vega 150”, with a capacity of 150 l (maximum temperature of 1,280 °C, with a rating of 10.0 kW and a tension of 230–400). All samples underwent the heating cycle and then different areas of each rib (including the gunshot wound edges and the concentric area of maximum 79 mm) were analyzed by a XRF device. One bovine rib was used as control sample, completely charred, and not subjected to the shooting test. For each sample, 2 or 3 measuring points were selected, one very near to the injury, one far and a possible third point in at intermediate distance for larger samples; one single area was measured in the control sample.

For the present application, a compact XRF spectrometer was used [20], mounting a MINI-X2 X-ray tube (Amptek, Bedford, MA, USA) with a maximum power of 4 W (50 kV, 200 µA) and a transmission rhodium anode. The X-ray detector used is a complete SDD spectrometer (XGL-SPCM-DANTE-25 model) from XGLAB Bruker Nano Analytics, Milan, Italy, with 17 mm^2^ active area, 500 m thickness and 12.5 m Be window. The working conditions of the XRF spectrometer for the measurements reported in this work were 40 kV and 0.06 mA with an acquisition time of 120 s each. Data reported in the following refers to peak areas normalized to the intensity of the Rayleigh peak of the Rh (tube anode) Kα line to keep into account the different geometry of our samples [21].

The XRF analysis focused on the main gunshot residues (Pb, Ba, Sb), but also other metallic residues were identified as discussed in the following. It is worth noting that the sensitivity of XRF spectrometers for GSR elements may vary according to their characteristic energy; for instance, in the present case, Pb has higher detection limits than Sb and mostly Ba, that means than smaller quantities of Pb give raise to a more intense peak. It is worth noting that Ba is the less significant among GSR, usually use to confirm the identification when present. Significant enough, Amadasi and co-authors [6] in some samples failed detecting Sb and/or Ba particles using SEM-EDX.

## Results and discussion

21 total measurements were made on the samples, as illustrate in Table 1; to this, sample 0, the un-shot rib, was considered as control sample. On this sample, presence of Ca and P were detected, coming from apatite, the main constituent of bone. In addition, Fe and Sr, a chemical vicary of Ca, were present, together with very small signals related to other chemical elements. Indeed, their presence can be linked either to diet, or other human behavior [22], or to a contamination of the sample from environment [14]. Furthermore, environmental contamination is a major issue that must be considered on dual levels. From an experimental point of view, contamination from environment could alter the information that can be obtained from the analysis due to previous use of the same oven. In terms of a real forensic scenario, contamination could play a significant role in the presence of additional traces from motor vehicles (car, bus, train, plane) and buildings with metallic structure [23, 24]. The finding of metal residues should also be considered in the differential diagnosis of those situations in which the metal particles found may mimic the explosion of a firearm, such as brake linings and their wear products [25, 26], the presence of fireworks [27–29], discharge residues from industrial tools and particles from occupational origins [30, 31].

In the following, the presence of GSR diagnostic elements, such as all other elements quoted, will be considered only when significantly higher than those present in the control sample.

As a general results, all the considered points show evidence of GSR; in the following, comparisons and evaluations will be carried out for the four classes of samples, i.e. NF, NL, DF and DL also in relation to the distance from the shooting wound.

**Fig. 2:**
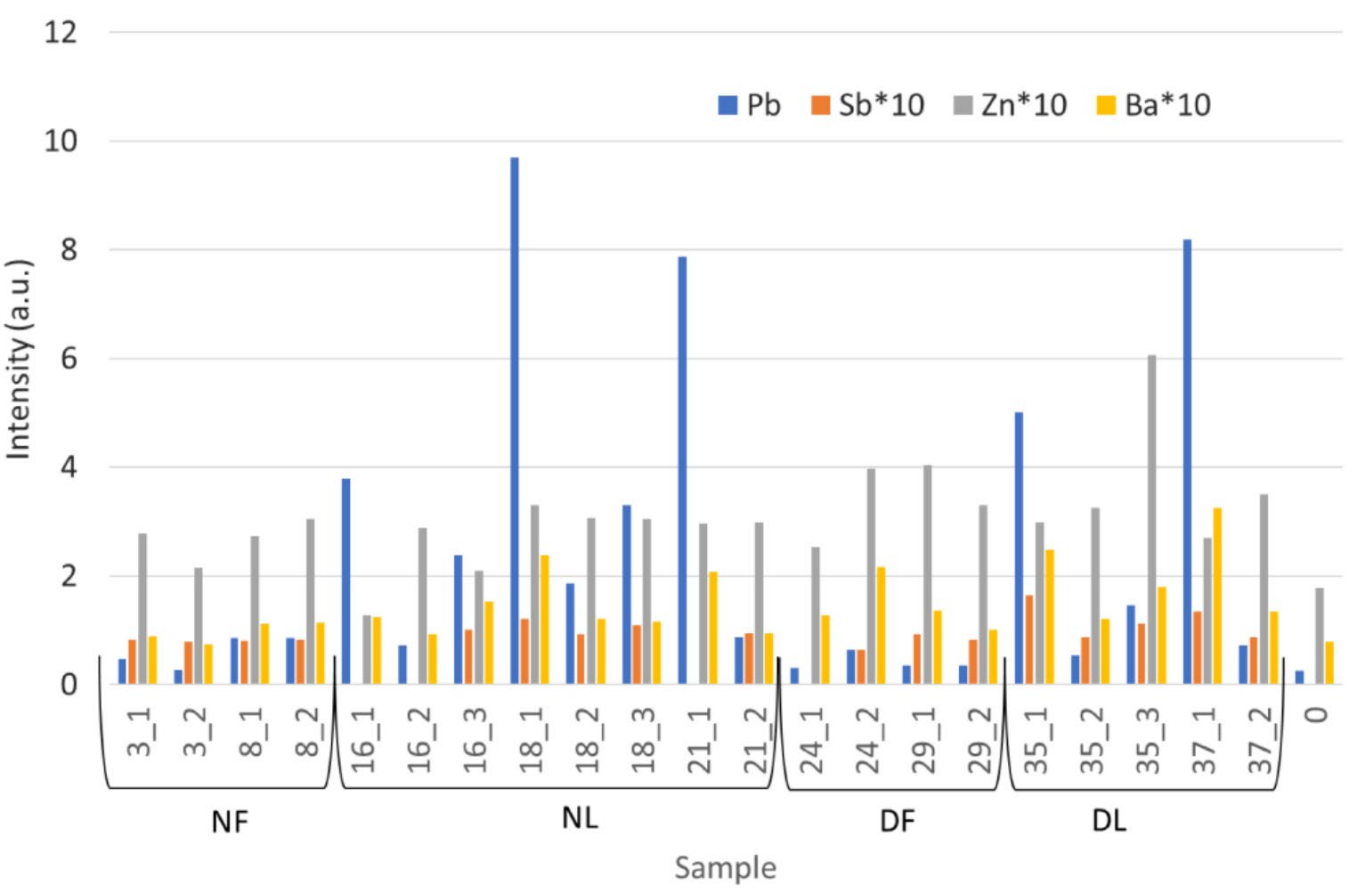
Results of XRF analysis on bone samples; for each measurement Pb, Sb, Zn, and Ba traces are reported

On the bases of the peak areas calculated for Pb, Sb, Ba and Zn in each spectrum, and normalized to the Rh anode emission [21], summarized in Fig. 2, we can outline our results as follows:

- NF: both samples (number 3 and 8) show presence of GSR with a small difference probably due to the different shape of the materials.
- NL: samples 16, 18 and 21 exhibit a yellow halo surrounding the entrance hole, mostly due to the lead styphnate subproduct deposition. In this aera, in fact, the Pb signal is higher, and slightly diminishes with distance, as Ba signals, being present also out of the yellow stain.
- DF: results for samples 24 and 29 are similar to those obtained for NF, even though not all the elements linked to GSR are always detected.
- DL: both samples (35 and 37) exhibit the yellow halo from the lead styphnate subproduct deposition even if the bones were shot before being skeletonized. Elements from GSR are well evident also far from the entrance hole, even if they diminish with the distance.

**Fig. 3:**
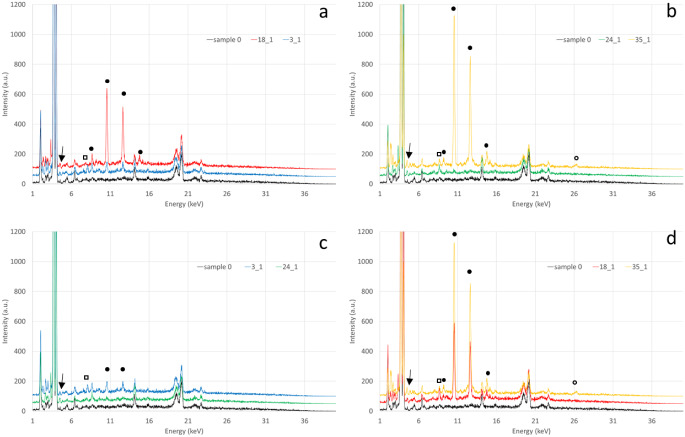
Spectra comparison: full metal jacket bullet GSR and lead bullet ones for naked ribs (**a**) and dressed ribs (**b**); naked and dressed bones for full metal jacket (**c**) and lead (**d**) bullets. Full dots indicate Pb signals, empty dots Sb, empty squares Zn and arrows indicate Ba peaks. Same sample spectra are drawn in the same color

Beside GSR, we also considered Zn presence, as it is significantly augmented in all samples. Indeed, Zn can derive from the bronze of the bullet, but also from the primer cup or from the primer mix [32]. Being the presence of Cu, also in bronze of the bullet, not so evident, we can assume the contribution of primer being predominant. Similar results had been obtained also when analyzing metallic residual by ICO-OES in control sample [14]. In some spectra (see spectra in Fig. 3) we also detected K and S signals, that can be present in primer mix materials [32].

Comparing full metal jacket bullet residuals with lead bullet ones for both naked and dressed ribs (see Fig. 2 parts a and b), GSR for lead bullets are much more evident in both types of ribs. On the other hand, comparing naked and dressed bones for full metal jacket and lead bullets respectively (see Fig. 2, part c and d) a small difference can be seen for the two types of bullets. Anyway, it is clear than lead unjacketed bullets GSR are well detectable in real cases (dressed ribs) by XRF conservative approach, while the detection of GSR for full metal jacket bullets is more critical. Similar results had been reached by Amadasi applying SEM-EDX [6] and ICP-OES [14].

As mentioned above, the forensic pathologist’s approach to charred bone remains is challenging: indeed, the effects of heat on soft tissues cause the loss of the ability to assess the injuries of the soft tissue itself. It also makes it difficult to differentiate between ante-mortal and post-mortal injuries caused by high temperatures. Cremating bones is a simulation of the most destructive event for a corpse and that the subsequent analysis of commingled and/or charred bone remains represents a huge challenge both in terms of identification issues and the study of the cause of death and investigation of the circumstances of death [33–35].

The use of XRF device for forensic purposes is not so widespread. However, recently Merelli et al. [15] simulated the interaction between a cadaver and a crime scene by placing skin samples on the ground of different workplaces and inside the trunk of a car, analysing samples with different approaches, including XRF device. Similarly, Berendes et al. [36] employed XRF analysis to search for GSR on fabrics, clothes, and other surfaces. Rosa et al. [16] investigated if blade chemical traces are transferred to defleshed bone tissue and if they remain there after a burning event. Tambuzzi et al. [17] studied the metal micro traces in electric marks in a case of death by electrocution (high-voltage current). There are only a few studies that used XRF devices aimed at searching for GSR particles [18, 19], however none applied it to charred bones. In this field of study, the importance of SEM-EDX is well known [1, 37–46]: It allows to obtain both morphological information and the elemental composition of GSR particles. However, this method is difficult to be accessed in forensic laboratory, expensive and time consuming. XRF spectroscopy is completely nondestructive and does not consume or damage the sample: this is an important feature because in forensic investigations specimens are often very small and the reproducibility of the analysis is pivotal in criminal proceedings. Nevertheless, XRF device is less sensitive than SEM-EDX to lighter elements and only supplies compositional information. The findings of this study underscore the potential of XRF as a screening tool. Further, more in-depth aspects will have to be investigated in subsequent studies, which will have to analyze a larger number of samples.

## Conclusions

These preliminary results demonstrate the potential use of XRF device in the detection and analysis of GSR in charred bones. Specifically, it could be used as GSR screening tools on burned and fragmented bone. In particular, we demonstrated how GSR particles survived to thermal destruction at very high temperatures, such as those reached in a furnace. Further, the analysis carried out in the most realistic situation of flesh covered bones showed how lead unjacketed bullets GSR are well detectable while the detection of GSR for full metal jacket bullets is more critical. The introduction of the XRF instrument in forensic ordinary practice could provide many advantages: less costs than SEM-EDX, possibility of analyzing many samples in a short time, without any sample preparation or alteration, allowing to perform an elevate number of replicable analyses.

**Table 1:** List of samples subjected to analysis by XRF device. Samples from each of the four categories were subjected to two or three measurements at the indicated distance from the gunshot hole. NF = “naked” ribs, hit by a full metal-jacketed bullet, NL = “naked” ribs, hit by a lead unjacketed bullet, DF = “dressed” ribs, hit by a full-metal jacketed bullet, DL = “dressed”, hit by a lead unjacketed bullet

Type	Sample	Position (mm)
NF	3_1	0
	3_2	60
	8_1	0
	8_2	47
NL	16_1	0
	16_2	75
	16_3	23
	18_1	0
	18_2	50
	18_3	18
	21_1	0
	21_2	67
DF	24_1	0
	24_2	68
	29_1	0
	29_2	60
DL	35_1	0
	35_2	79
	35_3	26
	37_1	0
	37_2	40

## Data Availability

Data availability possible on motivated request to the corresponding author.
